# A network psychometric analysis to advance the understanding of children’s physical literacy

**DOI:** 10.1186/s40359-026-04490-w

**Published:** 2026-04-06

**Authors:** Yucui Diao, Sitong Chen, Clarice Martins, Isaac Estevan, Lei Wang, Rongbin Yin, Cuixiang Dong, Lisa M Barnett

**Affiliations:** 1 School of Physical Education and Sport, Soochow University, No.50, Donghuan Road, Suzhou, Jiangsu province 215021 China; 2 Institute for Health and Sport, Victoria University, Melbourne, Australia; 3 Research Centre in Physical Activity, Health and Leisure, and Laboratory for Integrative and Translational Research in Population Health, University of Porto, Porto, Portugal; 4 AFIPS Research Group, Department of Teaching of Physical Education, Arts and Music, University of Valencia, Valencia, Spain; 5 School of Physical Education, Shanghai University of Sport, Shanghai, China; 6 School of Physical Education and Health, East China Normal University, No. 500, Dongchuan Road, Shanghai, 200241 China; 7 Institute for Physical Activity and Nutrition (IPAN), School of Health and Social Development, Deakin University, Melbourne, Australia

**Keywords:** Network psychometrics, Complex system, Self-report, Physical literacy, Topology

## Abstract

**Background:**

Despite growing interest in children’s physical literacy (PL), limited research has examined the relative importance of its constituent elements from children’s own perspectives using network-based methods. This study employed network psychometrics to identify the key elements (i.e., nodes with high centrality in the PL elements network) of PL based on children’s self-perception.

**Methods:**

A total of 1,520 Chinese children (752 girls, 49.5%; 768 boys, 50.5%), aged 7 to 12 years (*M* = 9.6 ± 1.7) completed a PL self-report (the Physical Literacy in Children Questionnaire). Regularized psychological networks were constructed to estimate the interactions between PL elements. Centrality was assessed using the expected influence (EI) index to identify potentially the most influential elements in the PL network. Network comparison test evaluates whether network structure, global strength, and centrality indices differed by sex and age groups (7–9 years and 10–12 years).

**Results:**

The elements with high EI values in the PL networks were *engagement and enjoyment* (psychological domain, EI = 1.64–2.22), *collaboration* (social domain, EI = 1.34–1.64), *self-regulation-physical* (psychological domain, EI = 1.12–1.63), and *speed* (physical domain, EI = 1.10–1.17). The PL network structure and global strength were invariant, though the specific elements with higher EI values differed slightly by sex and age groups.

**Conclusion:**

This study identified *engagement and enjoyment*, *collaboration*, *self-regulation-physical*, and *speed* as key elements of children’s PL. These findings support a child-centered, systems view of PL and highlight elements that merit further investigation in longitudinal or experimental research. Our findings empirically support the inclusion of social elements and children’s voices into PL assessment and promotion.

**Supplementary Information:**

The online version contains supplementary material available at 10.1186/s40359-026-04490-w.

## Background

Physical literacy (PL), as a novel idea to promote human physical activity (PA) and health, has gained prominence in global public health, PA, education, and sports science discourse over the past 15–20 years [1–3]. Rooted in its universal, holistic, and multidimensional nature [4], PL was defined by the International Physical Literacy Association as “*the motivation*,* confidence*,* physical competence*,* knowledge*,* and understanding to value and take responsibility for engagement in physical activities for life*” [5]. This seminal definition has served as a cornerstone for subsequent conceptual developments, though different countries have culturally reconceptualized PL in different ways. Despite its variations, common domains of PL have been identified, such as physical, psychological, cognitive, behavioral, and social domains [6–8]. These align with the Australian Sports Commission’s definition, which positions PL as a lifelong holistic learning journey that integrates physical, psychological, social, and cognitive abilities to lead healthy and fulfilling lives through movement and PA [9, 10]. This comprehensive conceptualization is the theoretical framework for this study.

However, despite the holistic definition of PL, there is no consensus regarding the importance of its constituent elements [8]. Theoretically, these elements are conceptually interconnected [4, 11], and equally important for PL development [10, 12]. Yet, this theoretical view contrasts with both practical intervention and stakeholder perceptions. In practice, efforts to promote PL in children often place disproportionate emphasis on its physical elements (e.g. motor skills) while giving less attention to cognitive, psychological, and social elements [13]. Furthermore, stakeholders’ priorities diverge from the theoretical view and from each other. For instance, Australian primary teachers prioritized *movement skills* (physical domain), *engagement and enjoyment* (psychological domain), *relationships* (social domain), and *safety and risk* (cognitive domain) [14], while Irish stakeholders emphasize *PA* (behavioral domain), *fundamental movement skills* (physical domain), and *enjoyment* (psychological domain) [15]. Conversely, stakeholders in England assign lower priority to physical elements [16]. Notably, these perspectives are predominantly adult-derived and may misrepresent children’s intrinsic developmental experiences and priorities. As PL development is an individualized process and children have the right to be heard in matters that concern them [17], understanding the relative importance of PL elements from a children’s perspective is essential.

Children’s perspectives, representing their experiences, perceptions, and understanding within their life-world [18], remain critically underexplored. The limited existing evidence suggests their views may differ from adult stakeholders’, with one qualitative study highlighting *motivation* and *confidence* (psychological domain) as the most important PL elements [19]. More fundamentally, merely collecting subjective ranking from children does not capture the complex interactions among PL elements [20]. Without modeling these interactions, any subjective prioritization remains incomplete or misleading, inevitably hindering the development of effective interventions and valid assessment tools [21]. Therefore, advancing a child-centered approach is necessary to move beyond collecting subjective preferences and instead identifying the importance of PL elements based on their empirical interactions.

Given the complex interactions among various elements in PL over time [1], it can be conceptualized as a complex system [22]. A complex system perspective recognizes that PL emerges from dynamic interconnections among its components. However, prevailing approaches relying on subjective rankings or traditional statistical models (e.g., factor analysis) are unsuitable to model such a system as they typically assume linearity and local independence, thereby failing to capture the synergistic interactions that define PL [23]. In contrast, network psychometric analysis provides a paradigm shift better suited to capture such complexity [24], conceptualizing psychological constructs as systems of interacting elements, represented as nodes (i.e., variables) and edges (i.e., statistical relationships) in a network [25].

This approach has been successfully applied to model interconnected systems in areas such as psychopathology and attitude, demonstrating its utility in revealing the structure of complex psychological constructs [26, 27]. This data-driven approach can identify key elements (i.e., centrally influential elements within a network) and comparing network structures, moving beyond subject ranking [28]. Understanding which PL elements are most influential within the system may help prioritize intervention targets. A preliminary network study has been conducted with preschoolers’ perceived PL (aged 4–6), identifying *speed*, *engagement and enjoyment*, *confidence*, *tactics*, and *collaboration* as key elements, with findings varying by sex and age group [21]. However, the key elements of primary school children’s PL, and how these may differ by sex and age group, remain unexplored.

Despite growing interest in PL, three key gaps remain regarding the relative importance of PL elements. First, most research reflects adult perspectives rather than children’s lived experiences. Second, existing studies rely largely on subjective rankings of PL elements. Third, few studies model the interconnections among PL components. Addressing these gaps requires a child-centered, systems-based approach. Thus, the first aim was to identify the key elements (i.e., nodes with high network centrality) within the self-perceived PL network of primary school children using network psychometric analysis. The second aim was to examine how PL’s network structure varies by sex and age groups. To our knowledge, this is the first study to apply network psychometrics to primary school children’s self-perceived PL. By modelling interactions among perceived PL elements, this study identifies the most influential components from children’s perspectives. The findings will provide empirical evidence for viewing PL as a complex system and offer potential insights for developing child-centered interventions and assessments.

## Method

This cross-sectional study examined the network structure of self-perceived PL among primary school children in China.

### Participants

Eight provinces were selected from northern (Shandong, Hebei, and Shanxi province) and southern (Zhejiang, Hubei, Hunan, Fujian, and Guangxi province) China, capturing regional socioeconomic and educational diversity. Eighteen schools from these provinces (four primary schools in Shandong province, two primary schools in each of the seven remaining provinces) were recruited based on convenience and accessibility. A total of 2,160 children and their parents agreed to participate in this study (consent rate = 87.8%). During data cleaning, we first excluded 65 children who were out of the predefined age range (7–12 years). Subsequently, we used listwise deletion to remove incomplete cases with missing data (missing household registration data, *n* = 377; missing out-of-school sports training data: *n* = 369; incomplete responses on the PL-C Quest: *n* = 155; missing BMI, *n* = 84), ensuring analytical accuracy. The final complete dataset included 1,520 children (female, *n* = 752; 49.5%) aged 7 to 12 years (*M* = 9.6, *SD* = 1.7). The study protocol and procedures were approved by the institutional review board of Shanghai University of Sport [No. 102772021RT071].

Demographic variables (age, sex, BMI, grade, age group, residence) showed no significant differences across the analytical, total, and excluded samples except for region (northern vs. southern China), which differed significantly between the analytical sample and both the total and excluded samples (Table 1). To assess potential bias introduced by missing data, we compared key demographic characteristics between the analytical and excluded samples. The absence of significant differences in most variables suggests no systematic bias with respect to these characteristics. Although the out-of-school sports training differed between analytical and excluded samples (*p* < 0.05), children from southern China had a significantly higher sport participation rate than their northern counterparts in the total sample (46.7% vs. 35.5%; *χ²* (1) = 22.94, *p* < 0.001). This indicated that the difference in out-of-school sports training between the analytical and excluded samples likely reflects this underlying regional characteristic rather than a selection bias. Given that the complete-case analysis (*n* = 1520) remained large and broadly representative on key demographics (i.e., sex and age group), multiple imputation was not implemented. Nonetheless, the regional difference warrants caution when generalizing findings to specific provincial contexts.

**Table 1:** Demographic characteristics of the samples and the comparisons between analytical and excluded samples

Characteristic	Full sample/ (*n* = 2160)^*^		Analytical sample (*n* = 1520)	Excluded sample/s *n* ^*^		Test statistic	*p* ^b^
Age, *M* (SD)	9.50 (1.7)	*n* = 2160	9.55 (1.7) ^a1^	9.40 (1.8)	*n* = 640	*t* = 1.776	0.076
BMI, *M* (SD)	17.0 (3.1)	*n* = 2076	17.0 (3.1) ^a2^	17.0 (3.3)	*n* = 556	*t* = -0.332	0.740
Sex, *n* (%)		*n* = 2160			*n* = 640	*χ*^2^ = 0.025	0.875
Male	1089 (50.4)		768 (50.5) ^a3^	321 (50.2)			
Female	1071 (49.6)		752 (49.5)	319 (49.8)			
Grade, *n* (%)		*n* = 2160			*n* = 640	*χ*^2^ = 5.888	0.317
1	331 (15.3)		227 (14.9) ^a4^	104 (16.3)			
2	339 (15.7)		233 (15.3)	106 (16.6)			
3	377 (17.5)		256 (16.8)	121 (18.9)			
4	408 (18.9)		286 (18.8)	122 (19.1)			
5	356 (16.5)		257 (16.9)	99 (15.5)			
6	349 (16.2)		261 (17.2)	88 (13.8)			
Age groups, *n* (%)		*n* = 2095			*n* = 575	*χ*^2^ = 4.384	0.036
7–9 years old	1035 (49.4)		731 (48.1) ^a5^	306 (53.2)			
10–12 years old	1060 (50.6)		789 (51.9)	269 (46.8)			
Residence, *n* (%)		*n* = 1783			*n* = 263	*χ*^2^ = 1.153	0.283
Urban	1296 (72.7)		1112 (73.2) ^a6^	184 (70.0)			
Rural	487 (27.3)		408 (26.8)	79 (30.0)			
Out-of-school sports training, *n* (%)		*n* = 1791			*n* = 271	*χ*^2^ = 12.232	< 0.001 ^#^
Yes	741 (41.4)		655 (43.1) ^a7^	86 (31.7)			
No	1050 (58.6)		865 (56.9)	185 (68.3)			
Region (China), *n* (%)		*n* = 2160			*n* = 640	*χ*^2^ = 60.872	< 0.001 ^#^
Northern	928 (43.0)		735 (48.4) ^a8^	193 (30.2)			
Southern	1232 (57.0)		785 (51.6)	447 (69.8)			

Note. ^*^ Some variables have missing values, so the sum does not reach the sample size. ^a^ test for difference between analytic sample and full sample. ^a1^ *t* test: *p* = 0.433; ^a2^ *t* test: *p* = 0.895; ^a3^ *χ*^2^ test: *p* = 0.948; ^a4^ *χ*^2^ test: *p* = 0.962; ^a5^ *χ*^2^ test: *p* = 0.404; ^a6^ *χ*^2^ test: *p* = 0.761; ^a7^ *χ*^2^ test: *p* = 0.318; ^a8^ *χ*^2^ test: *p* = 0.001(significant after Holm- Bonferroni correction). ^b^ test for difference between analytic sample and excluded sample. ^#^ significant after Holm-Bonferroni correction

### Physical literacy measure

As part of the same study, children were assessed from April 2023 to May 2023 using the 30 elements from the Physical Literacy in Children Questionnaire (PL-C Quest) Chinese version [29]. The PL-C Quest was selected because it captures multidimensional self-perceived PL in children. The scale consists of 30 elements in four domains [10]: physical (assessing motor skills and fitness acquired through movement), psychological (assessing attitudes, emotions, and their impact on an individual’s confidence and motivation to move), social (assessing interpersonal and environmental interactions related to movement), and cognitive (assessing the understanding of how, why and when to move). Each element contains two pictures, with the picture on the left representing a ‘more developed’ PL level and the image on the right representing a ‘less developed’ PL level.

For younger children in Grade 1 and Grade 2 (approximately 7- to 8-year-olds), their perceptions of PL were assessed one-on-one in a room with an interviewer reading out each scenario. Children were asked to make two dichotomous choices for each element. For example, to evaluate item 18 in the psychological domain for young children, the evaluator told the children that “*Some children feel they like being active in lots of different ways*,* because they enjoy it*” (by pointing to the picture on the left of the page), but “*Other children do not feel like being active in lots of different ways*,* because they don’t enjoy it*” (by pointing to the picture on the right of the page). Then the children were asked “*Which is more like you?*”. After the child pointed out the picture appropriate for him/her, the child was asked “*Is this picture A LOT like you*” (by pointing to the larger circle below their chosen picture) “*or A BIT like you*” (by pointing to the smaller circle below their chosen picture). The child then proceeded to complete each item in the PL-C Quest using the same dichotomous two-stage process. For older children in Grades 3 to 6 (approximately 8- to 12-years-old), a supervised-group administration was used; the administrator guided children by reading each scenario out loud and displaying the related images (20–35 students per group) through a self-completion process.

For scoring, the options for the ‘more developed’ picture were ‘a lot like me’ (assigned a score of four) or ‘a bit like me’ (three points), while the options for the ‘less developed’ picture were ‘a bit like me’ (two points) or ‘a lot like me’ (one point). Accordingly, perceived PL for each element was rated on a 4-point scale. Scores for each element were summed into the overall PL and subdomain scores (overall: range 30–120; physical: range 12–48; psychological: range 7–28; social: range 4–16; cognitive: range 7–28). The instrument has good test-retest reliability at two-week intervals (*r* = 0.71–0.90), internal consistency reliability across all four domains (polychoric α = 0.81–0.94), structural validity (CFI = 0.954, TLI = 0.950, RMSEA = 0.042), and measurement equivalence across sex and age groups among Chinese children aged 4 to 12 [29].

### Data analysis

Descriptive statistics and PL network estimation and comparison between sex and age groups were calculated using IBM SPSS version 26 (IBM Corp., Armonk, NY, USA) and RStudio version 4.1.3 (R Core Team, 2018), respectively. This study was in line with the reporting standards for psychological network analyses in cross-sectional data [30].

#### Descriptive statistics

The sample characteristics (sex, age groups, residence, etc.) were presented using descriptive statistics. Because PL scores were negatively skewed (-0.15 to -0.85), nonparametric Mann-Whitney U tests were used to examine the differences in the overall PL score and each subdomain by child’s sex (girls/boys), age groups (7–9 years/10–12 years), residence (urban/rural), out-of-school sports training (yes/no), and region (northern/southern). These comparisons were calculated for descriptive purposes only, and *p*-values were adjusted using the Holm-Bonferroni method.

#### Estimation method and visualization of network

The package qgraph [31] was used for network estimation and visualization. In line with current guidelines [32], we estimated a Gaussian graphical model (GGM) [33] for all PL elements. Given the ordinal nature of the data, the network was estimated using a polychoric correlation matrix (appropriate for ordinal data) [34], which approximates associations between underlying continuous latent variables. This approach had been justified by the robustness of GGM estimation with continuous data for ordered categorical data [35]. The model was based on the polychoric correlation matrix Network estimation was performed by combining the graphical lasso (glasso) with the extended Bayesian information criterion (EBIC) [36]. The tuning parameter γ was set 0.5 as per common practice to balance network specificity and sensitivity. We quantified the global connectivity in the network by the mean absolute edge weight and density [23]. These metrics should be interpreted cautiously due to the regularization inherent in EBIC glasso networks, which shrinks small partial correlations to zero and may affect the apparent network connectivity.

#### Centrality estimation

Centrality measures could be used to estimate the position and role of nodes in a network. In psychological networks, these metrics help identify nodes that have considerable influence over others [23]. This study used expected influence (EI), which integrates both positive and negative connections in a network, to quantify the relative influence of each element in the PL network [37]. EI values were standardized into Z-scores. A threshold of ≥ 1.0 was used to identify key PL elements, an exploratory criterion adopted from a prior network study of PL [21]. This threshold is not a strict statistical cutoff but a heuristic based on prior research. Higher EI values indicate a node’s greater influence on network activation and persistence [37]. Given the absence of standardized criteria for identifying key elements and the complexity of PL elements, this threshold (EI Z-scores ≥ 1.0) could enhance methodological representativeness.

#### Network comparisons

The network comparison test package [38] was used to assess global and local network invariance between sex and age groups through a permutation test with 1,000 iterations. Global network comparison included network structure (i.e., the overall patterns of connections) and global strength (GS; the absolute sum of all edge weights). Local network comparison focused on EI centrality to identify specific nodes that exhibited differential connectivity patterns between groups. Holm-Bonferroni correction of p-values were used due to multiple tests [38]. Age groups (7–9 vs. 10–12 years) were defined based on Piaget’s stages of cognitive development [39] and the Chinese physical education curriculum, which introduces more complex movement skills in later primary school [40].

#### Network accuracy and stability

To ensure the robustness of the estimated network, we used the package bootnet to assess centrality accuracy and stability [32]. First, the edge accuracy of the PL networks was estimated by calculating the bootstrapped confidence intervals (CIs) (95% CIs, *n*boots = 1000), using non-parametric bootstrapping. Narrower CIs indicated more accurate edge estimation. Second, the centrality stability (CS) coefficient was calculated using case-drop bootstrapping (*n*boots = 1000), which was the maximum drop in proportions to retain highly correlated (*r* > 0.7), with a minimum cut-off of 0.25 and ideally surpassing 0.5 [34]. Third, bootstrapped difference tests were conducted between centrality indices of the nodes (i.e., EI values) to test the stability of elements in the PL networks.

## Results

### Sample and demographics

Table 1 displays the participant characteristics of the full sample, analytical sample, and excluded sample. Just under half lived in northern China (48.4%) and around half were boys (50.5%) aged from 7.0 to 12.8 years (*M* = 9.6, *SD* = 1.7). Mann-Whitney U tests with Holm-Bonferroni correction (Table 2) showed boys rated themselves higher than girls in physical domain (*Z* = 3.50, *p* < 0.001), cognitive domain (*Z* = 3.19, *p* < 0.001), and the overall PL score (overall: *Z* = 3.66, *p* < 0.001). Younger children reported higher physical scores compared to older children (*Z* = -3.72, *p* < 0.001). But all effect size were small (*r* = 0.06–0.10). Descriptives and comparative results according to the area of residence, sport practice, and region are reported in supplementary Table 1.

**Table 2:** Overall physical literacy and subdomain scores for all children, and by sex and age groups

Domain	Total (*n* = 1520)		Girls (*n* = 752)		Boys (*n* = 768)			Girls VS Boys			7–9 years (*n* = 731)		10–12 years (*n* = 789)			7–9 years VS 10–12 years		
	M(SD)	Mdn	M(SD)	Mdn	M(SD)	Mdn		Z	*p*	*r*	M(SD)	Mdn	M(SD)	Mdn		Z	*p*	*r*
Physical (range 12–48)	35.1 (6.4)	35.0	34.6 (6.0)	34.0	35.6 (6.8)	35.5		3.50	< 0.001^a^	0.09	35.7 (6.2)	35.0	34.5 (6.5)	34.0		-3.72	< 0.001^a^	0.10
Psychological (range 7–28)	22.4 (3.9)	22.0	22.1 (3.9)	22.0	22.6 (4.0)	23.0		2.42	0.016	0.06	22.4 (3.8)	23.0	22.3 (4.0)	22.0		-1.05	0.878	0.03
Social (range 4–16)	13.0 (2.4)	13.0	12.8 (2.5)	13.0	13.2 (2.3)	13.0		2.47	0.014	0.06	13.1 (2.4)	13.0	13.0 (2.4)	13.0		-1.27	0.205	0.03
Cognitive (range 7–28)	23.0 (3.7)	23.0	22.7 (3.6)	23.0	23.2 (3.7)	23.0		3.19	0.001^a^	0.08	22.9 (3.7)	23.0	23.0 (3.6)	23.0		0.71	0.478	0.02
Overall (range 30–120)	93.5 (13.8)	93.0	92.3 (13.2)	91.5	94.6 (14.2)	95.0		3.66	< 0.001^a^	0.09	94.1 (13.6)	94.0	92.9 (14.0)	93.0		-1.61	0.107	0.04

Note. Mean (*M*); standard deviation (*SD*); Median (*Mdn*). Mann–Whitney U tests were used to for all group comparisons. Effect size *r* was calculated as r =$\:Z/\sqrt{N}$, where N is the total sample size (1520) for each comparison. ^a^ significant after Holm-Bonferroni correction

### Physical literacy networks estimation

**Fig. 1:**
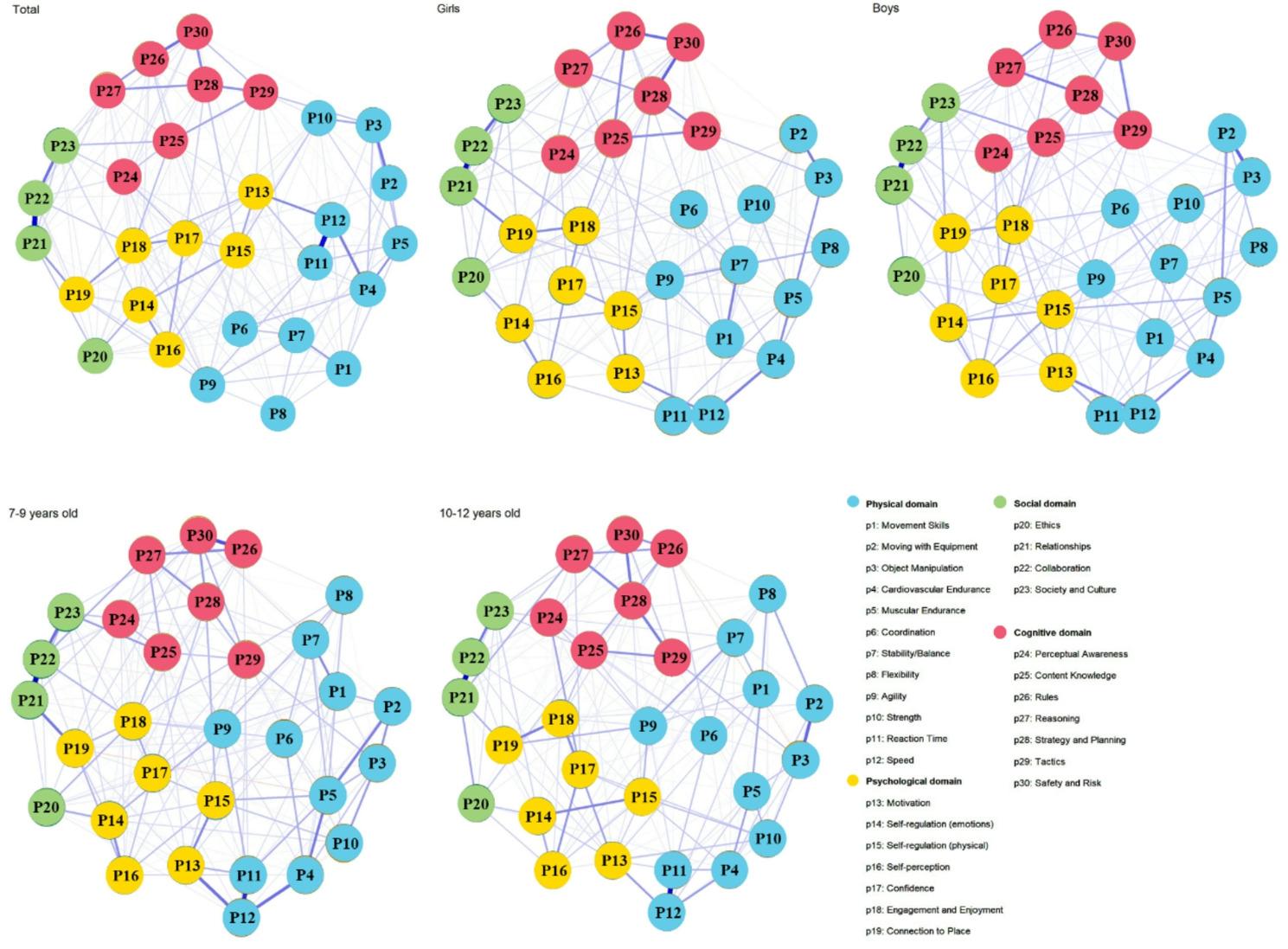
EBIC-glasso networks of physical literacy for all children (*n* = 1520), and by sex (girls, *n* = 752; boys, *n* = 768) and age groups (7–9 years, *n* = 731; 10–12 years, *n* = 789). *Note*. Nodes represent PL elements and edges represent regularized polychoric partial correlations between elements. Edge thickness indicates the strength of the partial correlations and edge color indicates the correlation valence (purple = positive; red = negative). Blue indicates physical domain elements, yellow indicates PL psychological domain elements, green indicates PL social domain elements, and fuchsia indicates PL cognitive domain elements

The regularized network included all 30 PL elements, yielding 435 possible edges in the total sample and by sex and age groups. The densities of the networks range from 0.455 to 0.524 (total: 0.506; girls: 0.471, boys: 0.487, young children: 0.524, old children: 0.455). The mean weights of non-zero edges were 0.031 (for the total sample, girls, and young children) and 0.032 (for boys and old children). The plot for all children (Fig. 1) shows that the elements within the same domain tended to cluster together, except *ethics* (P20), which occupies a position between the psychological and social domains. The positive edge weights between *relationships* (P21) and *collaboration* (P22) within the social domain, as well as between *reaction time* (P11) and *speed* (P12) within the physical domain, are the highest.

### Centrality estimation

**Fig. 2:**
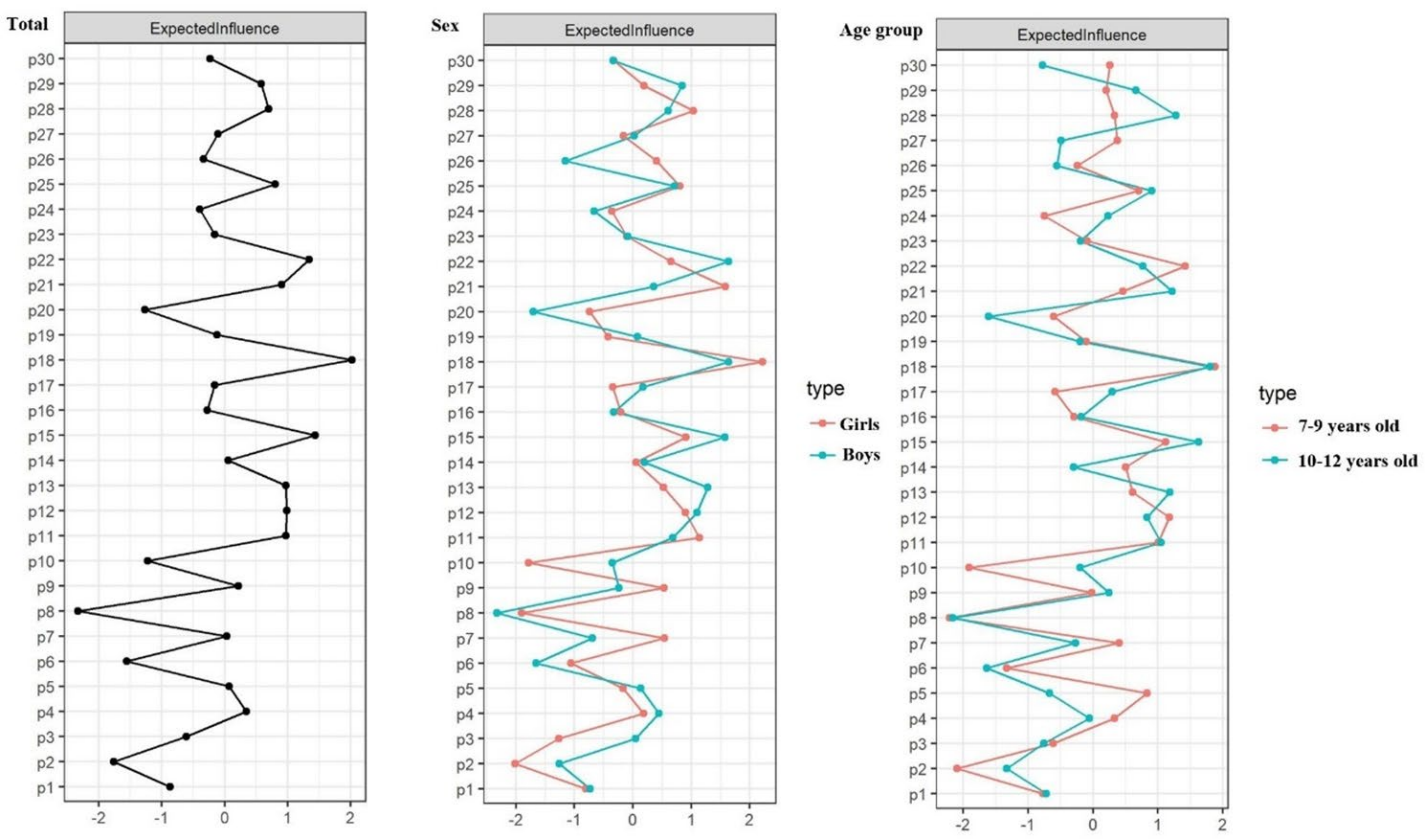
Standardized centrality estimates of physical literacy elements for all children (*n* = 1520), and by sex (girls, *n* = 752; boys, *n* = 768) and age groups (7–9 years, *n* = 731; 10–12 years, *n* = 789). *Note*. P1: Movement skills; P2: Moving with equipment; P3: Object manipulation; P4: Cardiovascular endurance; P5: Muscular endurance; P6: Coordination; P7: Stability/Balance; P8: Flexibility; P9: Agility; P10: Strength; P11: Reaction time; P12: Speed; P13: Motivation; P14: Self-regulation (emotions); P15: Self-regulation (physical); P16: Self-perception; P17: Confidence; P18: Engagement and Enjoyment; P19: Connection to place; P20: Ethics; P21: Relationships; P22: Collaboration; P23: Society and culture; P24: Perceptual awareness; P25: Content knowledge; P26: Rules; P27: Reasoning; P28; Strategy and planning; P29: Tactics; P30: Safety and risk

For the total sample (Fig. 2 total), *engagement and enjoyment* (P18) had the highest EI values (2.02), followed by *self-regulation-physical* (P15, 1.44) and *collaboration* (P22, 1.34). Specifically, *engagement and enjoyment* (P18) maintained high EI values across all subgroups (1.64 to 2.22). *Self-regulation-physical* (P15) exhibited high EI values in all subgroups (1.12 to 1.63), except for girls. *Collaboration* (P22) showed high EI values in boys (1.64) and young children (1.42), while *relationships* (P21) showed high EI values in girls (1.58) and older children (1.22). Additionally, *reaction time* (P11) had high EI values in all subgroups (1.00 to 1.14) except in boys, whereas *speed* (P12) had high EI values in boys (1.10) and young children (1.17). *Motivation* (P13) had high EI values in boys (1.28) and older children (1.18), while *strategy and planning* (P28) had high EI values in girls (1.03) and older children (1.28). *Flexibility* (P8) showed the lowest EI values relative to other elements in all groups (-2.16 - -2.34).

### Physical literacy networks comparison

No significant differences were observed in PL network structure between boys and girls (*M* = 0.194, *p* = 0.201) or between younger and older children (*M* = 0.202, *p* = 0.126). Similarly, GS did not differ significantly by sex (*GS*_girls_ = 13.96, *GS*_boys_ = 13.86, *S* = 0.10, *p* = 0.760) or age group (*GS*_7−9 years old_ = 14.11, *GS*_10−12 years old_ = 13.90, *S* = 0.12, *p* = 0.476). At the local network level, no elements showed significant difference in EI values by sex or age group (all *p* > 0.05).

### Physical literacy networks accuracy and stability

First, the bootstrapping 95% CI for most edges was relatively narrow in all groups (supplementary Fig. S1), suggesting good precision in the estimation of regularized partial polychoric correlations and indicating adequate stability for network comparison. Second, the CS coefficients of EI values (supplementary Fig. S2) all exceeded 0.5 in all groups (the total sample: CS = 0.75, 95% CI: 0.672, 1.00; girls/boys, CS = 0.75, 95% CI: 0.672, 1.00; young children: CS = 0.67, 95% CI: 0.594, 0.750; old children: CS = 0.75, 95% CI: 0.672, 1.00), indicating adequate reliability of the identified key elements and the robustness of subsequent group comparisons. Finally, the results of centrality difference test are presented in supplementary Fig. S3.

## Discussion

The relative importance of PL elements, and more critically, the nature of their interactions, remains poorly defined, particularly from the children’s perspective. To address this gap, we utilized network psychometric analysis to move beyond identifying important elements in isolation and to instead investigate the network of interactions between PL elements in children. Our main findings showed that the key elements of PL for children aged 7–12 years are *engagement and enjoyment* (psychological domain), *collaboration* (social domain), *self-regulation-physical* (psychological domain), and *speed* (physical domain). The network structure, GS, and EI values of PL elements were invariant across sex and age groups. To our knowledge, this is the first study to examine the relative importance of PL elements and their network structure patterns from the perspective of children in elementary school (aged 7–12 years).

### Physical literacy key elements for children

The child-centered analysis (Fig. 2 Total) identified psychological (*engagement and enjoyment*, *self-regulation-physical*) and social (*collaboration*) elements as central to PL, whereas physical elements such as *flexibility* demonstrated the lowest EI values. This was contrary to adult stakeholders’ motor skill-centric emphasis in PL [14, 15]. This divergence highlights that children’s intrinsic psychological needs (e.g., enjoyment) and social dynamics (e.g., peer collaboration) may be more pivotal for their PL than the motor skills predominantly prioritized by adult stakeholders. This finding is supported by a recent study which aimed to derive a short version of the PL-C Quest. That study prioritized which elements to retain based on theoretical and data driven decisions and scored elements according to these criteria. Authors reported that in the physical domain, the elements reflecting motor skills scored poorly [41].

Specifically, *engagement and enjoyment* (psychological domain) reflects individual’s positive emotions and experiences derived from movement and PA [10]. Its higher EI value across all subgroup networks suggest that *engagement and enjoyment* is a potent activator and a central hub in children’s self-perceived PL network, as it likely forms reinforcing feedback loops with other elements. Enjoyment may be associated with increased PA participation, thus enhancing physical competence and interaction with peers, and success in these domains, in turn, generating more enjoyment and sustained engagement in PA. This is consistent with both Self-Determination Theory, which posits that enjoyment fosters intrinsic motivation for PA [42, 43], and empirical studies identifying enjoyment or affective responses as one of the most robust predictors of PA behaviors [44, 45]. Thus, as a critical precursor to PA participation [46, 47], *engagement and enjoyment* has powerful influence within children’s self-perceived PL network.

*Self-regulation-physical*, the psychological ability to recognize and manage physical signals (i.e., fatigue and exertion) [10], is measured in the PL-C Quest through a hill-climbing scenario contrasting strategic pacing (i.e., planned pacing) with impulsive exertion. By combining bodily awareness and cognitive planning, this element directly embodies physical-cognitive-psychological integration. Critically, such an effortful and deliberative self-regulation process reflects an individual’s self-control competence [48, 49], which is associated with higher PA levels [50, 51], less sedentary behavior [52], and better physical and mental health [53] in children and adolescents. Thus, its high centrality in children’s self-perceived PL networks is consistent with this evidence. Interestingly, it emerged as a key element of PL for boys but not for girls in our network analysis. This suggests that while self-regulation is important, its relative centrality in the girl’s PL network may be surpassed by other elements (e.g., *strategy and planning*). One possible explanation is that girls generally have higher self-control competence than boys [54]. This observed difference is preliminary and warrants further investigation to confirm its robustness and explore the underlying reasons.

*Collaboration* reflects the social skills for successful interaction with others [10]. According to the social interaction theory, children’s learning and development are facilitated through their interactions with others [55, 56], a process where peers and friends play a crucial role [57, 58]. In fact, peer support has been identified as a more important influence on children’s PL than parental and teacher support [59]. Critically, our network analysis provides empirical support for the social function by revealing the strongest edge in the PL network between *collaboration* (P22) and *relationships* (P21). This indicates that in children’s minds, the ability to collaborate is inextricably linked to the ability to form positive relationships. These two elements form a synergistic pair rather than separate skills, strengthening one likely directly strengthens the other, thereby amplifying their combined impact on children’s self-perceived PL. Furthermore, in team games, children’s interactive collaboration not only enhances their social skills but also improves communication, cooperation, and problem-solving skills, physical competence, and fostering emotional and cognitive development. Therefore, *collaboration* and *relationships* have high centrality in children’s self-perceived PL.

Speed-related elements in the physical domain were key elements of children’s self-perceived PL across both sex (i.e., *reaction time* for girls and *speed* for boys) and age groups (i.e., *speed* for younger children and *reaction time* for older children). These two elements are all involved in sprint scenarios, being universally prevalent in children’s spontaneous play. In China, sprint running is mandated as a fundamental movement skill and speed-related physical fitness component within the physical education and health curriculum of elementary schools [40]. Consequently, children might be more inclined to evaluate their self-perception of physical competence through these speed-related movements, with which they are more familiar. According to competence motivation theory [60], children with higher self-perception of physical competence demonstrate increased motivation for participating in PA [61, 62], greater persistence in PA [63], and higher PA level [64], ultimately fostering better social interactions [65]. Thus, children with high speed-related perceptions are likely to experience more positive psychological outcomes and expanded social networks. This interplay underscores why speed-related elements have emerged as a key PL element for children.

*Strategy and planning* in the cognitive domain emerged as a key element of PL for girls and older children, but not for boys and younger children. For sex-based patterns, girls may rely more on cognitive strategies in their engagement in PA, possibly due to cognitive differences favoring executive functioning in girls and spatial reasoning in boys [66]. Additionally, societal norms historically frame sports as male-dominated, assuming boys naturally excel through physicality while girls are stereotyped as less assertive [67], with such gender-stereotyped attitudes linked to lower motor skill perceptions in girls [68]. Girls may thus be more likely to compensate for their weaknesses in motor competence through cognitive and strategic learning in sport.

For the age-based patterns, although *strategy and planning* had fewer connections in the older children’s network, its average connection strength was higher compared to the network of younger children (Fig. 1). This suggests that its role becomes more focused and powerful within older children’s self-perceived PL network, which aligns with developmental theory. Younger children (aged 7–9) are typically engaged in mastering fundamental movement skills [69] and exhibit concrete operational thinking (rule-based, tangible problem-solving) [70]. In contrast, older children benefit from maturing executive functions such as working memory and cognitive flexibility, which support more complex tactical thinking [71]. Thus, *strategy and planning* appears to integrate more powerfully with other competencies at this later developmental stage.

Additionally, *motivation* (psychological domain) is identified as a key element of PL in older children but not in younger ones, reflecting the growing role of children’s internal drives to be physically active. Older children (aged 10–12) commonly experienced both a decline in interest in PA [72, 73] alongside an increase in the complexity of movement skills. As their need for autonomy grows [42], *motivation* helps their sustain engagement in complex, self-regulated physical tasks (e.g., sport strategies). Consequently, older children may increasingly rely on *motivation* as a compensatory mechanism, bridging the gap between declining intrinsic interest and growing task complexity by enabling effort regulation. This process aligns with the increase importance of *self-regulation-physical* during this age group. Together, *motivation* and *self-regulation-physical* might form key psychological supports that help children maintain PA engagement at later childhood.

### Physical literacy network structure

The relatively high network density is consistent with the theoretical conceptualization of PL as a highly interconnected holistic construct. The consistent intra-domain clustering patterns observed in our networks aligned with prior latent variable modeling evidence (e.g., CFA-based structural validation) [29, 74–76], and confirm the theoretical domain structure of PL. However, our analysis crucially extends these findings by demonstrating the entire relational structure (i.e., the pattern of interactions between PL elements) is invariant across sex and age groups. Whereas traditional factor models confirm what elements belong together, our network approach demonstrates how they interact on a system level, demonstrating the methodology’s unique capacity to capture universal dynamics.

Notably, the network analysis identified elements that functioned as bridges between domains. A key finding was that *ethics* (P20, theoretically an element of the social domain) occupied a bridging position between the social and psychological domains. Its stronger functional coupling with psychological processes (e.g., *self-regulation-emotions*) than with other social elements suggests it may be a crucial link that integrates socio-emotional competencies within the PL network. This proximity likely arises from concurrent assessment of both elements through scenarios demanding emotional regulation, including ethical choices (e.g., post-game handshakes after loss) and affective control (e.g., reaction to dart-misses), thereby strengthening their functional coupling in children’s behavioral schema. This system approach initially identifies both highly (i.e., potential targets for intervention prioritization) and lowly influential elements via inter-element correlations that classical factor analysis cannot capture, underscoring network methodology as essential for operationalizing PL’s conceptual complexity.

Our network psychometric analysis empirically supports the conceptualization of PL as a complex system. The identification of psychological, social, physical, and cognitive elements as key elements across sex and age groups aligns with complex systems theory. These key elements exhibit high EI centrality that ensures network struct robustness [77], enabling PL to preserve its relational and stable structure despite sex and age groups. From an intervention perspective, targeting these key elements with high EI centrality capitalizes on their role as amplifiers that catalyzed the activation across the PL network [24]. Consequently, it is plausible to speculate that targeted modifications to central elements propagate systemic influence, thereby inducing system-wide optimization of PL networks. Nonetheless, this mechanism needs to be further explored using longitudinal data.

### Implications

Our study provides insights into the theoretical perspectives, assessments, and interventions related to PL. Theoretically, our findings reveal the complex network of interactions that constitute PL as a system. By identifying central elements and their interconnections within children’s self-perceived PL network, we uncovered the holistic structure of children’s PL across sex and age groups. This network-based and systems-oriented approach reconceptualizes PL as a complex system rather than a static trait, offering a methodological framework for assessing the relative importance of PL elements.

Regarding assessment tools, our findings indicate that social elements (i.e., *collaboration* and *relationships*) are key elements of PL from children’s perspectives, necessitating their formal integration into future PL conceptualizations and structural models. Moreover, future PL assessments should adopt a child-centered participatory framework, complementing traditional Delphi techniques with structured child consultations to enhance the ecological validity and developmental alignment of measurement tools. In terms of intervention design, the identified PL elements with high EI centrality may help prioritize targets for future systemic and holistic PL programs. It is important to note that these implications are derived from cross-sectional, self-perceived data and require validation through longitudinal and experimental research.

### Limitations

This study has some limitations. First, our cross-sectional design precludes causal inferences. Although a high EI value suggests that an element is centrally connected in a network, it does not have to establish that these elements with high EI values are developmental drivers or the most effective intervention targets. Also, the cross-sectional nature also means we cannot capture dynamic changes in PL networks over time. To validate this, longitudinal network studies are needed to examine causal dynamics and experimental studies are required to test the efficacy of interventions focusing on these key elements. Second, we did not examine the relationship between the key elements of PL with behavioral domains (such as PA), which could be examined in the future. Third, although self-report measures validly capture children’s self-perceived PL, they are subject to potential biases like social desirability or inaccurate self-assessment (common-method bias). Future studies could consider findings in relation to objective measures for the domains and elements where this is possible (e.g., physical competence assessments, cognitive tests) to examine whether differences exist in its key elements.

Fourth, the use of convenience sampling for school recruitment may limit the generalizability of our findings to the broader population of Chinese children. Approximately 18% of the total sample was excluded through listwise deletion due to missing key demographic variables (i.e., household registration, out-of-school sports training, BMI). Although the analytical sample did not differ significantly from the excluded or total samples by sex and age group, significant differences were found in region (see Table 1), indicating potential selection bias. Missing data, particularly for BMI, may also introduce bias, as children with missing BMI data may be more likely to be overweight/obese [78]. Such sample characteristics may also affect the representativeness of the sample and the stability of our network models, although the CS coefficients for centrality estimates exceeded the recommended threshold (0.67–0.75). Future research could implement standardized electronic data capture systems to minimize missing demographic variables. Fifth, the sample is limited to Chinese children, and cultural factors may influence perceptions of PL. Thus, findings may not be generalizable to other cultural contexts.

## Conclusion

This study identified *engagement and enjoyment*, *collaboration/relationships*, *self-regulation-physical*, and *speed* as key elements of children’s PL. By applying network psychometrics to explore children’s self-reported PL, our cross-sectional analysis generally revealed stable network structures across sex and age groups, although there were key elements with high EI values that varied slightly by sex and age groups. These results empirically illustrate the interconnected nature of PL as perceived by children and identify potential key elements that are statistically central within the network. The identified centralities reflect statistical relationships within a perceptual network and do not establish causal drivers of PL development. Consequently, our study provides a foundational, data-driven model that shifts the conceptualization of PL toward a complex systems framework. Future longitudinal and experimental studies are needed to test the temporal dynamics and causal efficacy of intervening on these central elements. For practice, our findings suggest that future PL assessments could be enhanced by more fully integrating the social domain and children’s voice, and that interventions might consider prioritizing elements highlighted by this child-centered network approach.

## Supplementary Information

Supplementary Material 1.

## Data Availability

The data that support the findings of this study are not openly available due to reasons of sensitivity and are available from the corresponding author upon reasonable request.

## Abbreviations

BMI: Body mass index
CI: Confidence Interval
CS: Centrality stability
EBIC: Extended Bayesian information criterion
EI: Expected influence
Glasso: Graphical lasso
GS: Global strength
PL: Physical literacy
PL-C Quest: Physical Literacy in Children Questionnaire
