# Supplementary material for: A network psychometric analysis to advance the understanding of children’s physical literacy
Source: BMC Psychol. 2026 Apr 6;14:756. doi: 10.1186/s40359-026-04490-w (PMC13188483; doi:10.1186/s40359-026-04490-w)

**Supplementary Figure S1.** Edge weight sampling bootstrap graph for all children (n = 1520), and by sex (girls, n = 752; boys, n = 768) and age groups (7-9 years, n = 731; 10-12 years, n = 789)


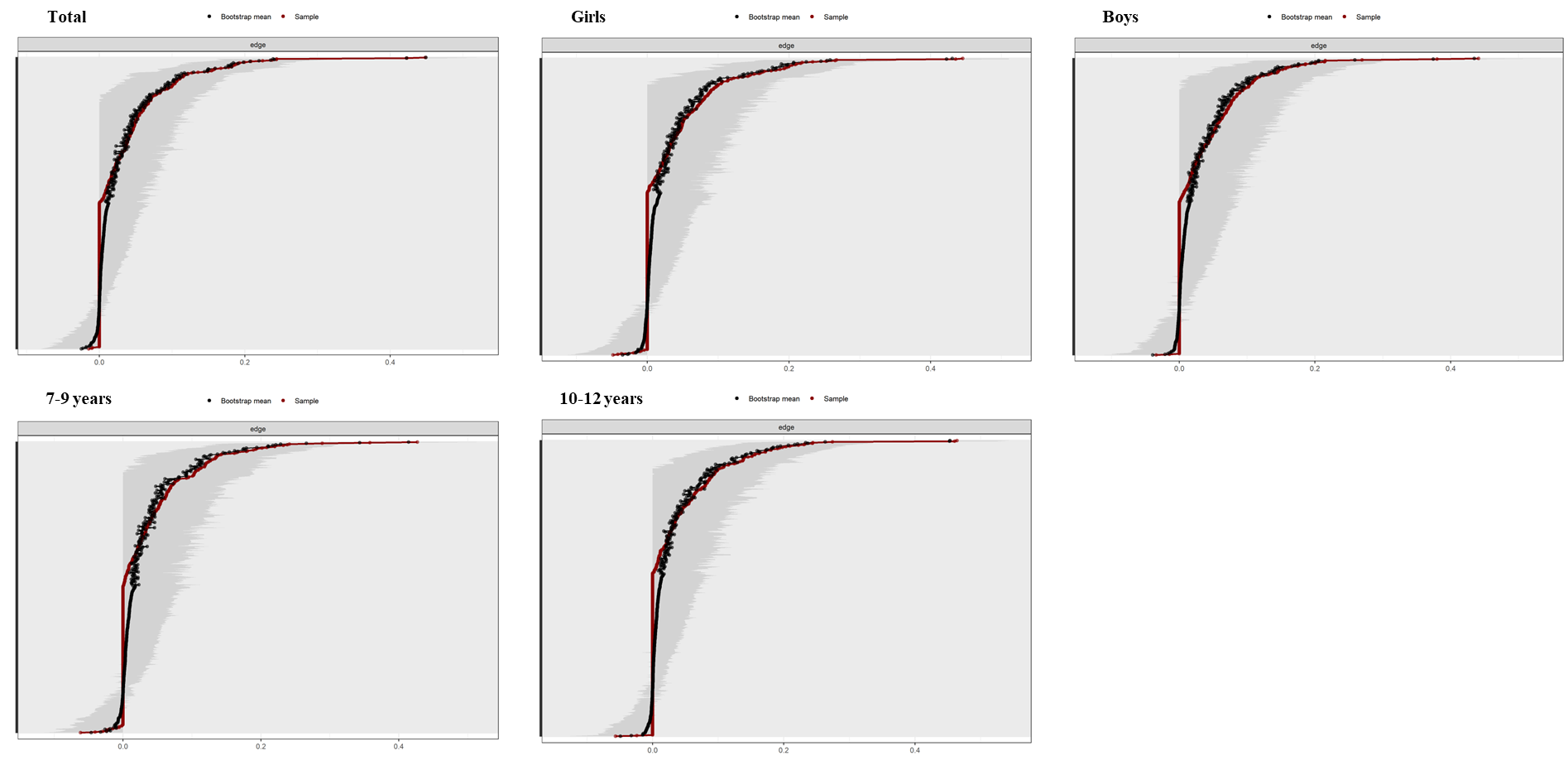

Supplement: Supplementary file 1 — Supplementary Material 1. [file 40359_2026_4490_MOESM1_ESM.zip › Supplementary Figure S1 Edge weight sampling bootstrap .docx]
