# Supplementary material for: A network psychometric analysis to advance the understanding of children’s physical literacy
Source: BMC Psychol. 2026 Apr 6;14:756. doi: 10.1186/s40359-026-04490-w (PMC13188483; doi:10.1186/s40359-026-04490-w)
Supplement: Supplementary file 1 — Supplementary Material 1. [file 40359_2026_4490_MOESM1_ESM.zip › Supplementary Table 1 Overall PL and subdomain scores_revised.docx]

**Supplementary Table 1.** Overall physical literacy and subdomain scores for all children, and by residence (urban *vs* rural), out-of-school sports training (yes *vs* no) and region (southern *vs* northern)

| Domain | Total  (n = 1520) | | Urban  (n = 1112) | | Rural  (n = 408) | |  | Rural VS Urban | |  | Yes  (n = 655) | | No  (n = 865) | |  | Yes VS No | |  | Northern  (n = 735) | | Southern  (n = 785) | |  | Southern VS Northern | |
| --- | --- | --- | --- | --- | --- | --- | --- | --- | --- | --- | --- | --- | --- | --- | --- | --- | --- | --- | --- | --- | --- | --- | --- | --- | --- |
|  | M(SD) | Mdn | M(SD) | Mdn | M(SD) | Mdn |  | Z | p |  | M(SD) | Mdn | M(SD) | Mdn |  | Z | p |  | M(SD) | Mdn | M(SD) | Mdn |  | Z | p |
| Physical  (range 12-48) | 35.1 (6.4) | 35.0 | 35.3 (6.5) | 35.0 | 34.7 (6.1) | 34.0 |  | -1.86^b1^ | 0.063 |  | 36.3 (6.3) | 36.0 | 34.2 (6.3) | 34.0 |  | 6.45 ^c1^ | 0.000^a^ |  | 34.7 (6.4) | 34.0 | 35.5 (6.3) | 35.0 |  | 2.68 ^d1^ | 0.007 |
| Psychological  (range 7-28) | 22.4 (3.9) | 22.0 | 22.5 (3.9) | 23.0 | 21.9 (4.1) | 22.0 |  | -2.08^b2^ | 0.037 |  | 23.0 (3.7) | 23.0 | 21.9 (4.1) | 22.0 |  | 5.00 ^c2^ | 0.000^a^ |  | 22.3 (3.9) | 22.0 | 22.4 (4.0) | 22.0 |  | 0.84 ^d2^ | 0.401 |
| Social  (range 4-16) | 13.0 (2.4) | 13.0 | 13.1 (2.4) | 13.0 | 12.8 (2.6) | 13.0 |  | -1.96^b3^ | 0.050 |  | 13.3 (2.3) | 13.0 | 12.8 (2.5) | 13.0 |  | 3.41 ^c3^ | 0.001^a^ |  | 13.0 (2.4) | 13.0 | 13.0 (2.4) | 13.0 |  | -0.10 ^d3^ | 0.918 |
| Cognitive  (range 7-28) | 23.0 (3.7) | 23.0 | 23.2 (3.6) | 23.0 | 22.5 (3.8) | 22.0 |  | -3.06^b4^ | 0.002^a^ |  | 23.5 (3.6) | 24.0 | 22.6 (3.6) | 23.0 |  | 5.52 ^c4^ | 0.000^a^ |  | 23.1 (3.6) | 23.0 | 22.9 (3.7) | 23.0 |  | -1.13 ^d4^ | 0.257 |
| Overall  (range 30-120) | 93.5 (13.8) | 93.0 | 94.0 (13.7) | 94.0 | 91.9 (13.9) | 91.5 |  | -2.61^b5^ | 0.009 |  | 96.1 (13.5) | 96.0 | 91.5 (13.7) | 91.0 |  | 6.54 ^c5^ | 0.000^a^ |  | 93.0 (13.5) | 93.0 | 93.8 (14.1) | 93.0 |  | 1.07 ^d5^ | 0.285 |

*Note*. Mean (M); standard deviation (SD); Median (Mdn). Mann – Whitney U test was used to calculate residence, out-of-school sports training, and region differences for the overall and subdomain scores separately. a indicates a significant difference after Holm-Bonferroni correction. B, c, and d present the effect sizes of the Mann-Whitney U test. b1: r = -0.05, b2: r = -0.05, b3: r = -0.05, b4: r = -0.08, b5: r = -0.07; c1: r = 0.17, c2: r = 0.13, c3: r = 0.09, c4: r = 0.14, c5: r = 0.17; d1: r = 0.07, d2: r = 0.02, d3: r = -0.01, d4: r = -0.03, d5: r = 0.03.
